# Parent Perspectives on Family-Centered Pediatric Electronic Consultations: Qualitative Study

**DOI:** 10.2196/16954

**Published:** 2020-04-09

**Authors:** Rhea Verma, Tamar Krishnamurti, Kristin N Ray

**Affiliations:** 1 Department of Pediatrics University of Pittsburgh School of Medicine Pittsburgh, PA United States; 2 Department of Medicine University of Pittsburgh School of Medicine Pittsburgh, PA United States

**Keywords:** consultation, referral, telemedicine, telehealth, child health, child health services

## Abstract

**Background:**

Electronic consultations, which use store-and-forward transfer of clinical information between a primary care physician and a specialist, improve access to specialty care. Adoption of electronic consultations is beginning in pediatric health care systems, but little is known about parent perspectives, informational needs, and preferences for interaction with this new model of care.

**Objective:**

This study aimed to examine parent perspectives about electronic consultations, including perceived benefits and risks, anticipated informational needs, and preferences for parent engagement with electronic consultations.

**Methods:**

We recruited caregivers of pediatric patients (aged 0-21 years) attending visits at an academic primary care center. Caregivers were eligible if their child had ever been referred for in-person specialty care. Caregivers participated in a semistructured interview about electronic consultations, including general perspectives, desired information, and preferences for parental engagement. Interviews were transcribed and qualitatively analyzed to identify parent perspectives on electronic consultations in general, information parents would like to receive about electronic consultations, and perspectives on opportunities to enhance parent engagement with electronic consultations.

**Results:**

Interviewees (n=20) anticipated that electronic consultations would reduce the time burden of specialty care on families and that these had the potential to improve the integrity and availability of clinical information, but interviewees also expressed concern about data confidentiality. The most detailed information desired by interviewees about electronic consultations related to data security, including data confidentiality, availability, and integrity. Interviewees expressed concern that electronic consultations could exclude parents from their child’s health care decisions. Interviewees saw value in the potential ability to track the consultation status or to participate in the consultation dialogue, but they were more ambivalent about the idea of read-only access to consultation documentation.

**Conclusions:**

Parents identified the potential risks and benefits of pediatric electronic consultations, with implications for communication with families about electronic consultations and for incorporation of features to enhance parent engagement.

## Introduction

### Background

The demand for pediatric specialty care exceeds supply, resulting in challenges such as long wait times for families seeking specialty care [1,2]. One innovative and promising strategy to improve timely access to specialty care is electronic consultations, a store-and-forward type of telemedicine [3]. Electronic consultations, also called eReferrals and eConsults in specific health systems, allow primary care physicians (PCPs) to communicate with specialists about a specific patient as a way to *right size* the patient’s specialty care [4]. In an electronic consultation, the PCP sends a clinical question to a specialist, along with photos, videos, and any other relevant media through a secure electronic platform. The specialist reviews the information at a later time and then sends recommendations to the PCP, including advice for further PCP-driven evaluation, potential management through the PCP, or timeframe for an in-person specialty consultation if indicated. The PCP, consequently, communicates those recommendations back to the patient. This process, intended for nonurgent specialist input, may completely avert the need for the patient to physically attend an in-person specialty consultation, or it may guide interval care so that evaluation and management can be optimized while awaiting in-person specialty consultation.

To date, electronic consultations have been implemented in several health care systems, including the Department of Veterans Affairs, the Mayo Clinic, San Francisco General Hospital, and the Los Angeles County Department of Health Services, and these show initial promise in their ability to efficiently meet the demand for specialty care [5-7]. After electronic consultations became a required preliminary step for all referrals through the LA County Department of Human Services, 25% of the electronic consultations were resolved without a specialist visit, and the percentage of referrals scheduled within 30 days improved from 24% to 30% [8]. Studies of clinician perspectives on electronic consultations identified potential clinician-perceived benefits and risks [9,10]. Studies of the perspectives of adult patients are more limited, but these studies reported general acceptability [11,12]. Patients appear to value the potential for electronic consultations to improve access to specialist expertise and to place PCPs in a more central role [7], but patients raise concerns that the information transmitted may not be comprehensive and that quality of the outcome may depend on patient-PCP relationships [11,13]. In addition, adult patients also expressed a desire to be more informed about and engaged with the electronic consultation process, which often appeared to occur without patient knowledge [13].

Electronic consultations are now also beginning to be used by innovative pediatric referral centers. The Canadian Champlain Building Access to Specialists through eConsultations eConsult service reported on over 1000 pediatric electronic consultations between 2014 and 2016, where 36% of the electronic consultations were resolved without an in-person visit and PCPs reported high satisfaction [14]. At Boston Children’s Hospital, a pilot electronic consultation from 2014 to 2016 significantly reduced wait time for specialty appointments and improved appointment completion rates, with PCPs also reporting improved communication and care [15]. For the adaption and implementation of new technology in general, input from the end user is needed to optimize acceptability and impact. When new technology and care models are directed at children, caregiver perspectives and preferences become an important part of the design processes, given the essential role of adult caregivers as integral partners of the pediatric care team [16]. Thus, as additional pediatric systems consider adopting electronic consultations, it will be important to consider parental views on electronic consultations and optimal design of electronic consultation systems, yet little is known about parent perspectives and preferences around this model of care.

### Objective

This study aimed to fill this knowledge gap by assessing parent perspectives, anticipated informational needs, and preferences to guide the development of family-centered electronic consultation systems. Specifically, we aimed to answer three questions: (1) what do parents see as the benefits and risks of electronic consultations? (2) what information would parents like to receive before a physician initiates an electronic consultation for their child? and (3) what value do parents perceive in features that could heighten parent engagement with electronic consultation systems? Answering these questions will facilitate a more family-centered design of pediatric electronic consultations to optimize usability and usefulness from parent perspectives.

## Methods

### Study Design

We performed a qualitative analysis of semistructured interviews to identify family preferences regarding electronic consultations for pediatric specialty care.

### Recruitment

During June 2019 and July 2019, we conducted semistructured interviews with caregivers of children attending primary care visits at the University of Pittsburgh Medical Center Children’s Hospital of Pittsburgh (CHP) Primary Care Clinic. The CHP Primary Care Center is an academic pediatric primary care center where the majority of patients are insured by Medicaid. Eligible participants were the parents of pediatric patients (aged 0-21 years) attending either well-child visits or acute visits whose child had ever been referred to specialty care. Pediatricians at the clinic identified potentially eligible participants and asked for permission for the research team to provide study information. A research team member then invited parents to participate in a semistructured interview and obtained verbal consent from participants. Participants received a US $25 gift card at the conclusion of the interview to compensate them for their participation. The University of Pittsburgh Institutional Review Board provided ethical review and determined this study to be exempt from formal further review.

### Interview Guide

An interview guide was developed to include questions that explored parents’ perspectives on electronic consultations, the information they would like to receive if electronic consultations were to be used for their child, and potential features to enhance family engagement. Electronic consultations were described for interviewees using standard language as a process involving the following:

A pediatrician summarizing a clinical question about a child in written form and sending it to a specialist. Sometimes, the written information could also include a picture or a video. The specialist then reviews this information, provides their recommendations, and sends it back to the pediatrician, who updates the family by phone within a few days.

**Figure 1:**
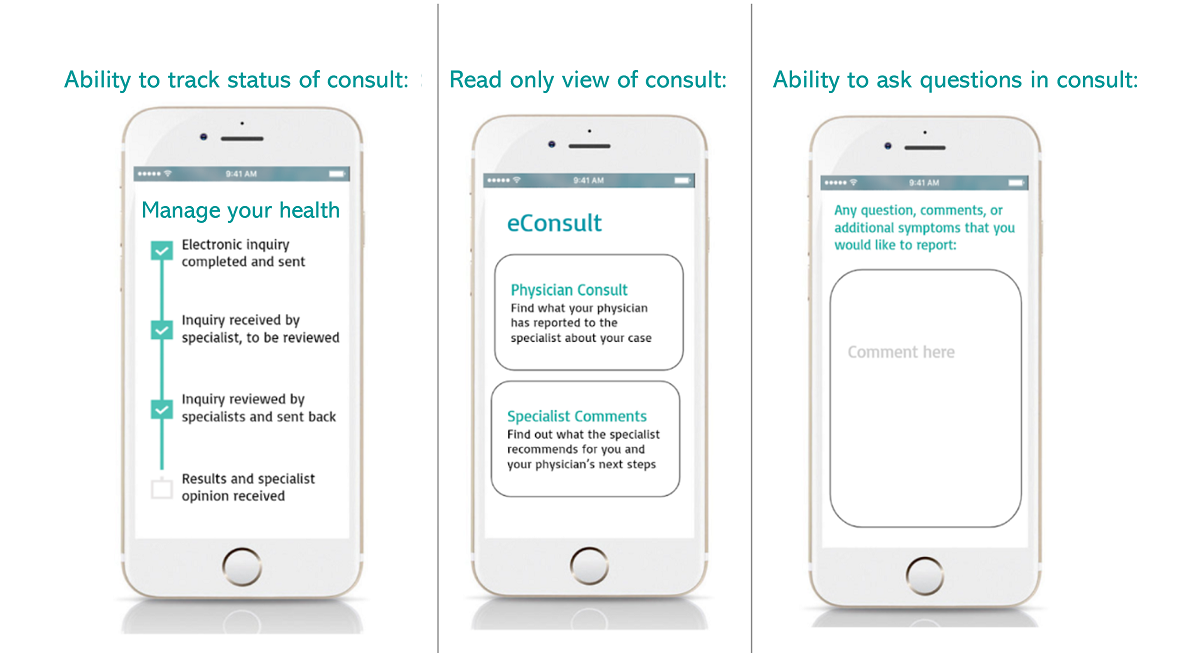
Prototype options for family engagement with electronic consultation. Three prototype static visual images sequentially presented to interviewees during interviews.

Interview prompts then asked about circumstances where they would might prefer to use either electronic consultations or in-person visits, information desired about the process, preferences regarding the information transferred within the electronic consultation, and experiences while accessing child health information on the internet (eg, through a patient portal). Finally, we examined parent perspectives on different levels of potential family involvement with the electronic consultation process. For this final question, we developed and presented static prototypes of three hypothetical options for family engagement with electronic consultations within a patient portal (Figure 1), with parents asked to discuss their reactions to each. First, parents were asked to consider an option where they could view the status of the electronic consultation (sent, read, or responded) but not the actual content of the consultations. Second, parents were asked to consider an option where they could read the actual text of the communication between the PCP and the specialist in a read-only view. Finally, parents were asked to consider an option where they could read the text of the communication between the PCP and specialist with the additional ability of adding their own questions and comments to the pediatrician-specialist electronic consultation dialogue.

### Qualitative Analysis

The interviews were digitally recorded and subsequently transcribed. Transcripts and audio files were stored without personal identifiers on a password-protected server. Two investigators (RV and KR) analyzed interview transcripts using thematic content analysis. After coding the first five interviews separately, a preliminary codebook was developed. Using this preliminary codebook, the two investigators then recoded the first five interviews and coded all subsequent interviews, meeting regularly to discuss discrepancies in coding and to refine the codebook to encompass additional emerging themes. Analysis was performed using qualitative data software (Dedoose, SocioCultural Research Consultants, Los Angeles, California), with recruitment continuing until thematic saturation was reached [17]. The results in this paper are organized around three major overarching themes: perspectives on electronic consultations in general, information parents would like to receive about electronic consultations, and perspectives on opportunities to enhance parent engagement with electronic consultations.

## Results

### Participants

In total, we interviewed 20 caregivers (17 mothers, 2 fathers, and 1 grandmother) of children referred to specialty care (Table 1). Reflecting clinic demographics, 90% (18/20) of the caregivers reported that their children were insured by Medicaid, and 85% (17/20) of the participants identified as black. All respondents reported owning a smartphone. In addition, 17 respondents reported experience using a web-based patient portal, either for themselves or for their child.

**Table 1:** Participant demographics.

Characteristics		Value, n (%)
**Participant age (years)**		
	<24	2 (10)
	25 to 44	16 (80)
	45 to 64	2 (10)
**Participant gender**		
	Female	18 (90)
	Male	2 (10)
**Participant relationship with patient**		
	Mother	17 (85)
	Father	2 (10)
	Grandparent	1 (5)
**Participant patient portal experience**		
	Portal user	17 (85)
	Nonuser	3 (15)
**Patient visit reason**		
	Wellness check	18 (90)
	Sick visit	2 (10)
**Patient insurance**		
	Commercial insurance	2 (10)
	Medicaid/Children’s Health Insurance Plan	18 (90)

### Perspectives on Positive and Negative Aspects of Electronic Consultations

In discussing electronic consultations as a potential substitute or complement to in-person specialty visits, interviewees discussed several ways in which electronic consultations could alter (1) the time burden of specialty care on families, (2) the transfer of information between the doctors and caregivers, and (3) caregiver involvement with the consultative process (Table 2).

There was general agreement that electronic consultations would reduce the time burden on families in multiple ways. Interviewees noted that if an issue could be resolved without a specialist visit, they would also be able to avoid unnecessary travel and reduce the time spent away from school and work. Interviewees were optimistic that electronic consultations might also reduce the time they currently spend waiting for specialist input in their child’s care:

I mean, I think that the wait time to get in to see a psychiatrist is like a year and a half. So if you have an issue and you need to get in there now, a digital consult might actually be helpful. That way, at least they can get you on some medicine or whatever.

Interviewees also expressed hope that if an in-person specialist visit was determined to be necessary, the electronic consultation process might still result in more efficient care if it could simplify scheduling processes or allow for increased efficiency because of previsit communication. For example, one mother suggested the following:

It would save time of sitting there and have me go over every [piece of medical history]—if you already had it when I come in, I can give you a brief synopsis of what it is and you can save a little bit of time there.

Interviewees had more mixed comments across issues related to information transfer. Interviewees expressed concern and hesitancy related to data confidentiality, stating concern that their child’s information would “get into the wrong hands.” Interviewees raising this concern elaborated that they would be particularly worried if multimedia information (eg, images) were included. Relatedly, some interviewees queried whether parental permission would be required for this digital transfer of information. However, interviewees also predicted a positive effect on data integrity and availability. Specifically, the ability of the specialist to have access to comprehensive health information was a feature in which many interviewees saw value. Interviewees also valued the potential “paper trail” of electronic consultations, which might clarify what information was available and who had access:

I think it would help out a whole lot. I think it will eliminate a lot of, you know, confusion and paperwork, too.

**Table 2:** Parent perspectives on anticipated benefits and risks of electronic consultations.

Theme		Definition	Example
**Time burden: potential benefits**			
	Less time to hear back	Hearing back electronically would allow faster access to specialist expertise	“I mean, I think that the wait time to get in to see a psychiatrist is like a year and a half. So if you have an issue and you need to get in there now, a digital consult might actually be helpful. That way, at least they can get you on some medicine or whatever.”
	Save unnecessary visits	Electronic consultation could avoid a need for specialty visits in some circumstances	“Yeah, so if they would’ve had [electronic consults], that would’ve saved us trips going to the hospital.”
	Save travel time	Time saved from not having to drive to specialty care location	“I guess that would be a little bit better for me, so I’m not traveling 20 million miles.”
	Less missed school/work/other commitments	Avoiding opportunity costs of missed school/work because of appointments	“I think that would make it a lot easier for some of us parents that have to deal with truancy.”
	Have a specialist agree that a visit is needed before going	Likes the idea of a specialist reviewing and agreeing that visits are needed rather than going in and finding out visits are not needed	“I’m sure she could have probably sent over, you know, the stuff from the bloodwork in conjunction with the growth chart in an e-mail or whatever and said, “Hey, what do you think of this?” And if it was medically necessary, then we’d go to visit.”
	Previsit communication can save time in overall appointment	Previsit consultation allows doctors to know what to look for	“So yeah, definitely the electronic way would have helped in the past and most likely in the future, as well, with them being able to see the issue prior to us getting there. So that way they can really relay to us exactly what’s going on.”
	Shorter appointments overall	Doctors know what to look for during appointments and have patient information ready	“it would save the time of sitting there and have me go over every—if you already had have it when I come in, I can give you a brief synopsis of what it is and you can save a little bit of time there.”
	Removes scheduling difficulty	Less stress about getting an appointment in a timely manner, especially if one is not needed at all	“I would have appreciated [an electronic consultation] versus me actually...going and schedule an appointment because it would have been the timeframe, the process...”
	Postvisit communication and follow-up are more timely electronically	Keeps postvisit communication concise, less need to keep going back in after the initial visit	“I definitely will like it when my child has an ongoing issue, you know, say, like asthma or something and they just need a refill. I don’t feel I should have to come into the office just for them to give me a refill. You know what I mean? Like just something that’s ongoing, you know?”
**Data availability, integrity, and confidentiality: potential benefits**			
	More comprehensive transfer of information	More comprehensive transfer of information from the primary care physician to the specialist	“But at least knowing that they have a heads up and they know what to look for and why we’re coming would be even more reassuring.”
	More convenient transfer of information	The transfer of information is more convenient when an electronic consultation is used	“I think it would help out a whole lot. I think it will eliminate a lot of, you know, confusion and paperwork, too.”
	Better paper trail	Clearer paper trail that parents can refer to as documentation of visit occurrence and visit content	“That’ll be kind of like my back-up, you know what I mean? My paper trail.”
**Data availability, integrity, and confidentiality: potential risks**			
	Data confidentiality	Data should not be discussed with unauthorized people	“Confidentiality, that’s it. That’s the only thing...I would be concerned about with stuff being sent electronically.”
	Incorrect/incomplete information transferred	Possibility of incomplete transfer of information or incorrect interpretation	“I think they have to see up close and personal because maybe there’s something that they can see that the picture didn’t quite capture, you know?”
	Inaccurate decisions made with incomplete information	Electronic consultations do not give the full picture and may result in incorrect diagnosis	“So if I didn’t bring her in we might’ve not found that. And it was good that we came in and we didn’t do it electronically.”
	More uncertainty when decisions are made with incomplete information	The specialist may not be sure how to diagnose based on just the information provided	“I’m like instead of just like ‘Oh yeah, you know, this *might* be the problem’ like no, I need you to look.”
**Parent involvement: potential risk**			
	Less parent interaction with specialist	Less direct interaction with specialists	“I need to ask questions, I need to know everything”; “I’m hands-on. I want to see you. I want you to physically see my child.”
	Reduced quality of communication	Less ability to ensure high-quality communication	“No, I’d rather go to a specialist and then hear it like from the horse’s mouth and then instead of being a third party, she can explain everything to me at that time.”
	Decreased opportunity to ask questions	Decreased parent involvement and opportunity to ask questions	“I would’ve preferred to just go in and see the specialist because it...it’s better. It – I mean, you get more answers that way, I guess, so.”
**Contextual factors impacting relative risk/benefit**			
	Family dependent	Interest in use of electronic consultations depends on family situation and preferences	“That one, too, it’s different because I guess it all depends on where people’s at within the medical field thing, because I feel that I can always ask the questions to them myself, not necessarily in between what they’re talking about.”
	Clinical situation dependent	The use of electronic consultations depends on the immediate clinical situation and the urgency for care	“If its more of an issue that’s more in-depth, where it actually has to be seen and they’re not too sure, then yes, I would prefer to just go, just to get more so a clear, a better answer to what’s going on.”
	Parent permission dependent	Parents should decide who can assess the patients’ records	“Of course, they get parental permission.”
	Anticipated value relative to current systems	Interest in electronic consultations expressed relative to current systems	“Yeah, that’s something I would be willing to use. It sounds like way more easier than the stuff that goes on now.”

Interviewees had mixed views on how accurate electronic consultations could be, which related to concerns about data integrity and availability. Specifically, interviewees stated with concern that if the specialists only receive the information that is specifically sent to them, they may miss the broader context of the child’s health.

An additional concern raised by some interviewees was that the process of an electronic consultation could exclude caregivers from the clinical conversations and decision making, in contrast to an in-person visit, limiting their ability to provide information and context, as well as their ability to ask questions:

I would’ve preferred to just go in and see the specialist because it...it’s better. It – I mean, you get more answers that way, I guess, so.

Balancing these advantages and disadvantages, overall, interviewees appeared to favor the possibility of using electronic consultations, with most interviewees stating that there are times that they would have preferred the use of an electronic consultation rather than an in-person visit to the specialist:

Yeah, that’s something I would be willing to use. It sounds like way easier than the stuff that goes on now.

However, interviewees noted that whether electronic consultations were appropriate in a given circumstance might vary with both the clinical indication as well as with family circumstances and comfort with technology:

Plus, I...I’m not computer illiterate, you know what I mean? So it’s OK for me.

### Desired Information About Electronic Consultations

When interviewees were asked about what information about an electronic consultation they would like to know when considering its use for their child, most interviewees suggested a desire for relatively detailed information, including what the steps of the consultation are, what information is being sent about their child, and the speed of the expected response:

I would like for them to explain the whole process. What it entails, what they’re going to – the information that they’re going to give the doctor and even the timeframe when they – when we should hear something. So yes, I would expect for them to explain everything before proceeding.

Some interviewees stated that they would want additional specific information, such as the rationale for use, specifics of information transfer and security, and potential outcomes of the process (Table 3). A small minority of interviewees, in contrast, expressed minimal need for information on the process.

**Table 3:** Parent perspectives on information they would like to receive at the time of pediatric electronic consultation.

Theme	Definition	Example
Minimal explanation desired	Parents want minimal explanation	“I think the process is pretty clear, and if it wasn’t, I’m sure I would be able to ask the questions electronically, so.”
Desire to know the speed of the consultation	Want to know when they will hear back from specialist	“I would like for them to explain the whole process. What it entails, what they’re going to – the information that they’re going to give the doctor and even the timeframe when they – when we should hear something. So yes, I would expect for them to explain everything before actually proceeding.”
Desire to know rationale for use	Parents want to understand why an electronic consultation is being used in their case instead of an in-person visit	“Why is it electronic consult versus seeing the person and...you know, actually in person.”
Desire to know what information will be transferred	Desire to know what information is being put in the consultation for the specialist to review	“I guess just letting me know everything that she was going to be doing, and you know, just keeping me informed of like, you know, I guess any pictures or videos or anything that’s being sent to them.”
Desire to know about security/quality of information transfer	Parents want to know what security measures are taken to protect the child’s information	“What kind of security is there? You know, if for some reason there would be some type of breach, what are the protocols to let the parents know that pictures of my child are no longer safe, that kind of thing. Those are the – probably the biggest questions in my mind.”
Desire to understand the steps/paper trail	Parents want to understand how information is being transferred and the steps to an electronic consultation	“Exactly what they’re taking the test on, like I need to go from point A to point B, C D. Every step that they’re doing, I need to know, and I need to be broken down.”
Explanation of possible diagnoses and management	Parents want possible diagnoses and treatments explained to them while awaiting consultation advice	“The most serious, the most important, and what can be helped – like a solution. So, yeah, that’s basically it. Like, the most serious, ‘This is this,’ like the most important about it, what can it affect, the stuff like that.”

### Perspectives on Parent Participation in Electronic Consultations

When presented with options that might enhance parent engagement with the electronic consultation process (Figure 1), almost all the respondents responded positively to the idea of a feature allowing tracking of the status of the electronic consultation (eg, sent, read, and replied) to help them know how the process was advancing and to anticipate when they might hear back (Table 4):

I guess I don’t have to keep calling people and them calling me back or saying, you know, they’re busy right now. I guess it would be plain in sight for me to be able to see myself instead of having to go through 50 people.

Interviewees were more ambivalent about read-only access to the electronic consultation. Some anticipated value in being able to “stay up to date” on the physician conversation, and others valued the potential to assess the quality of the physician-to-physician communication with a read-only feature:

Just to know what’s going on, and then determine if I want to see the consultant in person.

However, others were apprehensive that medical terminology would be confusing and reported they prefer to receive information verbally through their PCP after the electronic consultation process:

I would prefer they do that in private and then talk to me because I’m not no doctor and half the words that they going to be saying, I don’t know.

Relatedly, some interviewees also mentioned that getting too much information could be overwhelming.

Despite these concerns raised about reviewing electronic consultations through read-only access, interviewee responses were generally positive when asked about the possibility of a feature allowing parents to join the generalist-specialist dialogue by adding their own questions and comments to the consultation. Interviewees thought this feature would improve their ability to get questions answered and would also better approximate an in-person appointment:

If I have a question about something, I could ask the doctors directly, you know, see what they’re saying. ‘Cause that’s how it is when you go to appointments: they include you. So I would want to be included.

Many families also mentioned that if the doctor missed something or had incorrect information, this feature would give them the opportunity to correct them, which might consequently lead to a more accurate diagnosis. Two families, however, were skeptical of whether the parents’ comments would add value to a consultation between two doctors (“I don’t know on the medical perspective how it will be used”), and one caregiver interviewee suggested that a word limit should be placed to keep parent comments concise.

**Table 4:** Parent perspectives on parent participation in electronic consultations.

Theme		Description	Example
**Tracking electronic consultation status: benefit**			
	Ability to track consultation status	Benefit of tracking consultation status/reduces uncertainty	“I guess I don’t have to keep calling people and them calling me back or saying, you know, they’re busy right now. I guess it would be plain in sight for me to be able to see myself instead of have to going through 50 people.”
**Read-only access to electronic consultation documentation: benefit**			
	Ability to stay up to date	Ability to follow communication within the consultation	“...because you are in the loop, what’s going on, yeah. With this experience with [name] seven years ago, we wanted to know exactly was what going on every day or every time. Yeah, so I am pro to see the communication.”
	Ability to assess quality of electronic consultation	Ability to decide whether to trust the electronic consultation and the information given	“Just to know what’s going on, and then determine if I want to see the consultant in person.”
**Read-only access to electronic consultation documentation: risk**			
	May not understand medical terminology	Parents may feel excluded from interaction if they cannot understand terminology	“I would prefer they do that in private and then talk to me because I’m not no doctor and half the words that they going to be saying, I don’t know.”
	Parents would rather hear recommendations through a PCP^a^ than read them	Parent prefers for a PCP to explain the problem to them	“So I would prefer them do that in their time and then come explain it to me when they get, you know, all their facts and stuff together.”
**Ability to comment on electronic consultation dialogue: benefit**			
	Ability to get questions answered	Parent use of interactive features would increase timely answers	“I mean, once their initial communication is complete...at that time, once I view it, if I have a question or concern, I can type out a message and then send it out to both the doctor and the specialist.”
	Parent input/comments can improve consultation quality	Parent use of interactive features to add relevant information to improve the consultation quality	“Yes – that’s important as well too. Cause the parent has a different perspective. The parent might say, ‘OK, well, this is not what’s – its more geared toward this, this is the issue more’ or something. Year, I think that’s important too, to have that little, you know, open communication.”
	Parent input better approximates in-person visits	Parent use of interactive features would better replicate communication in an in-person visit	“Because if I have a question about something, I could ask the doctors directly, you know, see what they’re saying. ‘Cause that’s how it is when you go to appointments: they include you. So I would want to be included.”
**Ability to comment on electronic consultation dialogue: risk**			
	Skeptical that parent comments can add value	Parents worried that their comments may not be accounted for or may not be helpful	“I don’t know on the medical perspective how it will be used.”

^a^PCP: primary care physician.

## Discussion

Using a qualitative analysis of semistructured interviews, we identified caregiver perspectives on potential benefits and risks of electronic consultations in pediatric care, information desired by caregivers about electronic consultations before use, and reactions to potential strategies to enhance parent engagement with electronic consultations. With the adoption of electronic consultations just beginning in pediatric health care systems [14,15], these results are important for envisioning optimal parent engagement and proactively developing approaches to increase the acceptability, uptake, and impact of this emerging model of specialty care.

Caregivers appreciated a range of ways through which electronic consultations could reduce the time spent obtaining specialist expertise through in-person care. They anticipated benefits not only from gaining specialist advice more rapidly through this system but also from avoiding the time burden of scheduling and attending an in-person visit. These perceived benefits are supported by previous studies highlighting resolution of specialty care needs without in-person visits and improved time to appointment when visits are needed [8,15]. Although adult patients and primary care providers tempered similar perceptions with concerns about the electronic consultation process potentially adding delays to definitive care [9,13], parent interviewees did not voice such concerns. Overall, the perceived benefits in access by parents contributed to an overall positive perception of this model of care. Parents’ informational needs related to this domain were relatively straightforward, with a desire to know the anticipated timeframe for follow-up communication from their PCP.

Caregiver perceptions of risks and benefits related to data security (including data confidentiality, data integrity, and data availability) associated with electronic consultations were more varied. In general, parents raised concerns about electronic consultation data falling into “the wrong hands,” but they perceived benefits in the resulting “paper trail” regarding their child’s care. Parents also had mixed perceptions regarding whether the information transmitted between PCPs and specialists would be more or less comprehensive than current processes, with implications for their confidence in the accuracy of the resulting clinical decisions. Much of the information desired by parents about electronic consultations related to these data security concerns, including a desire to know what information will be transferred, who would have access, and details of data transfer security and quality. These concerns were not predominant features of previous studies of adult patients [13], perhaps suggesting a greater drive to be a good steward of data for others than for oneself. Alternatively, adult patient studies focused on individuals with experience using an electronic consultation system, whereas this study discussed a hypothetical system, it may be that these fears become allayed after the experience of using a well-designed system. Regardless, the study’s results suggest that, at least during the initial adoption phases, parents desire comprehensive information regarding all domains of data security (eg, data confidentiality, integrity, and availability) at the time of electronic consultation initiation. As a result, systems may wish to develop patient education tools so that the PCPs initiating electronic consultations can share this information accurately and efficiently.

Caregivers also voiced concerns about the idea that they would have less ability to contribute information or to ask questions of the specialist during the electronic consultation process. This relates to ideas of data integrity—information might be missing without caregiver involvement—but extends farther to the ways in which electronic consultations alter decision making in the triad of specialist, PCP, and parent. Adult patients who used electronic consultations appeared to value the strengthening of the role of their PCP relative to the specialist in clinical care [11], but this idea did not emerge from parent respondents considering hypothetical use of electronic consultations, who focused instead on the relative diminishing of their own role. This primarily suggests that centering the family-PCP relationship in the information about the electronic consultation process may improve parent acceptability. This also suggests that the opportunities to increase family involvement in electronic consultations may be particularly valued in pediatric settings.

Regarding specific strategies to increase family involvement, caregivers were generally interested in being able to track the electronic consultation process, with no risks of this strategy raised by parents. Parents were more ambivalent about having read-only access to electronic consultation dialogue, with some valuing the potential to ascertain accuracy but others expressing concern that it could further their sense of being outside of the process because of lack of comprehension or inability to participate. Of note, data from settings sharing clinical notes in general (eg, OpenNotes) suggest that patients do often identify accuracy concerns, but they also benefit from enhanced patient understanding and patient-doctor relationships [18,19]. Such findings suggest that read-only access to electronic consultations may not ultimately result in the disenfranchisement that some parents envision, but these parental concerns warrant consideration of other levels of engagement or actionable patient education to assuage these concerns. Specifically, parents preferred access to notes when access was paired with opportunities to add details and ask questions, effectively creating a three-way dialogue among parent, specialist, and PCP. Although caregivers perceived this to best approximate in-person visits, this strategy could actually be viewed as a step beyond usual care. Instead of sequential dyadic conversations (parent-PCP and parent-specialist), this option could generate an ongoing conversation among all three relevant parties.

This study has several limitations. First, interviews were conducted at a single, urban, and academic primary care center, and the majority of participants were African American and female. In addition, most participants reported experience using a patient portal either for themselves or their child, which differs from national estimates in which one-third of the individuals reported patient portal use [20]. All caregivers had children who were referred to specialty care, but the recency of that referral varied (some referred that day and some in the past). In addition, as formative work to inform the design of an electronic consultation system, this study asked caregivers to consider the hypothetical use of an electronic consultation system, and the findings may differ as parents gain experience with electronic consultations in general or systems with specific features. Finally, as a qualitative analysis, the study’s results should be considered hypothesis generating rather than hypothesis confirming.

In conclusion, the study’s results suggest that caregivers perceive value in the use of electronic consultations, largely motivated by more timely and efficient access to specialist expertise for their children. Parents wish to receive information about the confidentiality, integrity, and availability of clinical information throughout the electronic consultation process, and systems may also wish to include messaging about electronic consultations that centers the family-PCP relationship. Systems considering electronic consultation should consider developing clear communications that address parents’ concerns and informational needs before integration. The inclusion of design features to track the electronic consultation process and to contribute to a three-way dialogue was of interest to families. Incorporating these parent perspectives into the design of pediatric electronic consultations may enhance acceptability and uptake of electronic consultations and optimize their ability to improve upon current processes of specialty care delivery.

## Abbreviations

CHP: Children’s Hospital of Pittsburgh
NIH: National Institutes of Health
PCP: primary care physician
